# Projecting Range Limits with Coupled Thermal Tolerance - Climate Change Models: An Example Based on Gray Snapper (*Lutjanus griseus*) along the U.S. East Coast

**DOI:** 10.1371/journal.pone.0052294

**Published:** 2012-12-20

**Authors:** Jonathan A. Hare, Mark J. Wuenschel, Matthew E. Kimball

**Affiliations:** 1 NOAA NMFS Narragansett Laboratory, Narragansett, Rhode Island, United States of America; 2 NOAA NMFS Woods Hole Laboratory, Woods Hole, Massachusetts, United States of America; 3 GTM National Estuarine Research Reserve, Ponte Vedra Beach, Florida, United States of America; 4 Biology Department, University of North Florida, Jacksonville, Florida, United States of America; University of Hamburg, Germany

## Abstract

We couple a species range limit hypothesis with the output of an ensemble of general circulation models to project the poleward range limit of gray snapper. Using laboratory-derived thermal limits and statistical downscaling from IPCC AR4 general circulation models, we project that gray snapper will shift northwards; the magnitude of this shift is dependent on the magnitude of climate change. We also evaluate the uncertainty in our projection and find that statistical uncertainty associated with the experimentally-derived thermal limits is the largest contributor (∼ 65%) to overall quantified uncertainty. This finding argues for more experimental work aimed at understanding and parameterizing the effects of climate change and variability on marine species.

## Introduction

Temperature is a dominant factor shaping the distribution of marine species [1] [2]–[3]. This overarching effect results from the influence of temperature on a number of biological processes [4] [5]–[6]. Using the terminology of Fry [7], temperature is a lethal factor, a controlling factor and a directive factor. All animals have thermal limits above and below which death is rapid. Within these limits, temperature controls a number of rate processes including gene expression, enzyme kinetics, metabolism, activity, consumption, and growth. As a directive factor, fishes respond behaviorally, metabolically, and ecologically to changes in temperature; the best examples comes from seasonal migrations, which are distributional responses related to changes in temperature [8]. Fry’s [7] factors are analogous to the concept of the ecological niche [9], defined as a multidimensional space within which a species can persist; environmental and biological conditions define the dimensions [10]. Temperature is a dominant environmental factor determining the size and distribution of the niche of many fish species [11].

The relation between temperature, a host of biological processes, and species distributions leads to the expectation that species distribution will change in response to changing temperatures [12]–[13]. Seasonal migrations by fishes are common in temperate ecosystems [14] and variation in the timing of seasonal migrations has been linked to interannual variability in the seasonal cycle of temperature [15]. Inter-annual differences in fish species distributions are also common and in many cases have been linked to interannual temperature variability [16]–[17]. Further, an increasing number of studies document directional shifts in fish distributions and increases in abundance, and link these biological changes to climate variability and change [18]–[25].

In addition to observing changes in fish distribution and abundance in the recent past, studies are developing projections of changes in fish distribution and abundance under climate change [26]. Earlier studies were based primarily on atmospheric general circulation models, an understanding of atmosphere-ocean links, and documented environmental influences on fish population; these three pieces were combined conceptually to consider the effect of climate change on various fisheries [27] [28]–[29]. More recent studies used IPCC-class general circulation models, including coupled atmospheric-ocean dynamics, combined with biological models to project changes in abundance and distribution of fishes [30]–[34]. In all instances, with either conceptual links or coupled models, climate change is predicted to change the distribution of marine fishes; the magnitude of distributional changes depends on the magnitude of climate change.

One approach to projecting changes in species range is the niche-based model. This approach develops a statistical relationship between current distribution and environmental conditions. General circulation models, which project future environmental conditions, are then used to project future species distribution [35]–[36]. As a specific example, Cheung et al. [37] [30] developed niche-based models for more than 1700 species of fish and projected changes in distribution. The model of Cheung et al. [37] [30] also included dispersal and recently has been revised to include biogeochemical processes [38]. There are other specific approaches to niche based modeling [34], but the common theme is to statistically relate current species distribution to current environmental conditions and use this statistical relationship in climate projections.

Here we develop a different type of niche model, one based on a specific hypothesis that is supported by laboratory experiments and field observations. Our study species is gray snapper (*Lutjanis griseus*), which is a common coastal marine species found along the southeast coast of the United States. We hypothesize that the northern range is determined by overwinter mortality of juveniles in nearshore and estuarine nursery habitats [39]. We empirically link this hypothesis to the projections of general circulation models and predict changes in the northern limit of gray snapper along the east coast of the United States. We argue that the projections are directly relevant to the numerous other species with estuarine juvenile stages along the east coast of North America. Further, we believe that the general approach is applicable globally to coastal species whose ranges are limited by overwinter mortality. Finally, we identify the main factors contributing to uncertainty in our projections and outline future research necessary to reduce this uncertainty.

## Materials and Methods

### Background on Species

Gray snapper (*Lutjanus griseus*) is a reef associated, tropical species that occurs in the central Western Atlantic, extending from Florida, through the Gulf of Mexico and along the northern and central coast of Brazil [40]. Young-of-the-year gray snapper have been reported as far north as Massachusetts [14], but adults are rarely reported north of Florida [41]. Gray snapper make diel, seasonal and ontogenetic movements between habitats [42]–[44], as well as onshore-offshore movements related to spawning [43]. However, there is no evidence of large-scale, seasonal north-south movements that would allow juveniles from north of Cape Hatteras to move south and join adult populations off of Florida. Thus, it is likely that juveniles in northern estuarine habitats are expatriates and perish as water temperatures cool in the fall and early winter [45].

Here, we use chronic and acute thermal tolerance metrics for juvenile gray snapper reported by Wuenschel et al. [39]. They quantified the chronic threshold as the cumulative degree days below 17°C survived by juveniles in the laboratory under ambient decreasing fall temperatures. The value of 17°C is the physiological threshold for growth under ad libitum ration [46]. They quantified the acute thermal threshold as the lethal minimum temperature for juveniles exposed to a constant rate of temperature decline (3°C day^−1^).

### Overview of Modeling Approach

Our goal was to project the future northern limit of gray snapper based on the hypothesis that northern range is limited by thermal tolerance of juveniles during winter [39]. We also aimed to quantify uncertainty in our projections using different climate change scenarios, an ensemble of general circulation models, and including statistical uncertainty in the empirical relationships used to link species distribution to temperature. We develop an empirical link between air temperature and thermal tolerance metrics and then estimate the latitude where thermal tolerance will be exceeded in the future under different climate scenarios. Specifically, six steps were taken to develop the projections of species northern range and to quantify uncertainty and these are explained in detail below. The notation used for variables and equations through these six steps are summarized in Table 1.

**Table 1:** Summary of the notation used for variables and equations.

Symbol	Variable	Description
*i*	Climate model	14 climate models (see Table 2)
*e*	Estuary	12 estuaries (see Figure 1)
*y*	Year	
*k*	Climate scenario	3 scenarios: commitment, B1, and A1B scenarios
*t*	Time periods	3 time periods: 1980–2000, 2040–2060, and 2080–2100
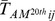	Mean minimum monthly winter air temperature for estuary *j* and climate model *i* estimate from the 20^th^ century model runs	
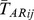	Mean minimum monthly winter air temperature for estuary *j* and climate model *j* from the NCEP/NCAR reanalysis	
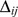	Mean bias correction for each climate model and estuary	
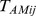	Modeled mean minimum monthly winter air temperature for estuary *j* estimated by climate model *i* in the future	
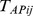	Projected (modeled – bias correction) minimum monthly winter air temperature for estuary *j* estimated by climate model *i* in the future	
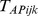	Projected minimum monthly mean air temperature from model *i* and for estuary *j* and climate scenario *k*	
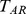	Minimum mean monthly winter air temperature from the NCEP/NCAR reanalysis combining data across estuaries and years	NCEP/NCAR grid cells matched to estuary locations. Only used continental grid cells in the comparison of NCEP/NCAR and estuarine temperatures
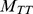	Thermal tolerance metrics for gray snapper including degree days <17°C and minimum daily winter temperature combining data across estuariesand years	data from daily temperature measurements in 12 estuaries over approximately 13 years [39]
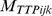	Projected thermal tolerance metrics for estuary *j*, model *i*, and climate scenario *k*	
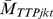	Ensemble average projection of thermal tolerance metrics for estuary *j*, climate scenario *k* and time period *t*	Ensemble average developed by averaging the projections of all 14 climate models for the given estuary, climate scenario, and time period
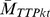	Ensemble average projection of thermal tolerance metrics for climate scenario *k* and time period *t*; data from each estuary is used to develop a predictive relationship for latitude (*Lat*)	
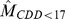	Estimated thermal tolerance for gray snapper relative to the cumulative degree days <17°C metric	[39]
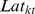	Latitude of a given thermal tolerance value for climate scenario *k* and time period *t*	
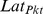	Projected latitude of northern range limit for climate scenario *k* and time period *t*	
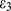	Unexplained error associated with the statistical relationship between 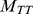 and 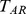 (eq. 3)	
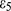	Unexplained error associated with the statistical relationship between and 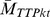 and 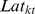 (eq. 5)	

### Step 1 General Circulation Models and Mean Bias Correction

The Fourth Assessment Report (AR4) of the Intergovernmental Panel on Climate Change (IPCC) [46] included simulations from 23 different GCMs run with standardized CO_2_ emission scenarios. Here we use 14 of these models (Table 2) and three emission scenarios: the commitment scenario, in which atmospheric CO_2_ is fixed at 350 ppm through the 21st century; the B1 scenario, in which CO_2_ increases to 550 ppm by the end of the 21^st^ century; and the A1B scenario, in which CO_2_ increases to 720 ppm by the end of the 21st century. The 14 GCMs were chosen because the results are publically available for the three climate scenarios (commit, B1, and A1B) and for a retrospective analysis of the 20th century (seen Table 2). Also, the models and scenarios included simulations through 2100. Some of the models have more than one run for one or more of the climate scenarios; only one run was included for each model and scenario to ensure that the models were treated similarly.

**Table 2:** List of general circulation models (GCMs) used in this study and their associated modeling centers.

Modeling Center	GCM
Bjerknes Centre for Climate Research, Norway	BCM2.0
Canadian Centre for Climate Modeling and Analysis, Canada	CGCM3(T47 resolution)
Centre National de Recherches Meteorologiques, France	CM3
Australia's Commonwealth Scientific and Industrial Research Organization, Australia	Mk3.0
Meteorological Institute, University of Bonn, GermanyMeteorological Research Institute of KMA, KoreaModel and Data Group at MPI-M, Germany	ECHO-G
Institute of Atmospheric Physics, China	FGOALS-g1.0
Geophysical Fluid Dynamics Laboratory, USA	CM2.1
Goddard Institute for Space Studies, USA	E-R
Institute for Numerical Mathematics, Russia	CM3.0
Institut Pierre Simon Laplace, France	CM4
National Institute for Environmental Studies, Japan	MIROC3.2 medres
Meteorological Research Institute, Japan	CGCM2.3.2
National Centre for Atmospheric Research, USA	CCSM3
UK Met. Office, United Kingdom	HadCM3

Three CO_2_ emission scenarios from 14 GCMs were used. Data were obtained from the Model and Data Group (M&D) at the Max-Planck-Institute for Meteorology (http://www.mad.zmaw.de/IPCC_DDC/html/SRES_AR4/index.html).

A given climate model may have systematic errors that result in an overall bias [26]. To correct for this bias, we compared air temperatures from the models’ 20^th^ century runs to observed air temperatures during the overlapping time period. For air temperature observations, we used the National Centers for Environmental Prediction (NCEP) and the National Center for Atmospheric Research (NCAR) reanalysis (http://www.esrl.noaa.gov/psd/data/gridded/data.ncep.reanalysis.derived.surface.html) [47]. This product combines observations and an atmospheric model to produce an even grid of atmospheric variables, in our case monthly mean surface air temperature with a spatial resolution of 2.5° latitude by 2.5° longitude.

Our goal was to forecast thermal tolerance criteria at 12 estuaries along the east coast of the United States (see below). For each estuary (*j*), the closest NCEP/NCAR grid cells were identified and these were then matched to the closest grid cell in each climate model (*i*). Minimum mean monthly winter air temperatures (December through March) were calculated for each year in the 20^th^ century model runs and in the NCEP reanalysis. Means from overlapping years (1948–2000, 1948–2001, or 1948–2005 depending on the climate model) were then calculated for the yearly mean minimum monthly winter temperature for the 20^th^ century model runs (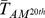) and for the NCEP/NCAR reanalysis (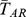) minimum over the overlapping years. The difference between these two means was defined as the mean bias correction (Δ*_ij_*) for the specific climate model (*i*) and estuary (*j*).
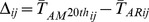
(1)


This value (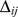) was used to adjust the air temperatures from the climate models (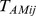) to correct for systematic model bias and to estimate the projected air temperature (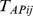) in each model (*i*) for each estuary (*j*).
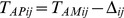
(2)


Understanding the cause of systematic model bias is an active area of research [48] [49–26]. A key assumption when applying a simple bias correction is that the bias is independent of the projected change [50]. We evaluated this assumption by calculating the correlation between bias and projected change for each model (*i*), climate scenario (*k*), and estuary (*j*). There were no significant correlations between bias and air temperature projections. There were negative trends between bias and latitude, but these trends were significant for only one of the fourteen climate models. These analyses indicate that the bias corrections are appropriate as a first order approach to remove systemic model bias from the long-term projections.

### Step 2 Relationship between Winter Water and Air Temperatures

The next step was to develop a statistical model to predict estuarine thermal tolerance thresholds from minimum monthly mean winter air temperature. The thermal tolerance metrics were cumulative degree days below 17°C and minimum daily winter temperature; these are the thermal tolerance criteria developed by Wuenschel et al. [39]. The winter air temperature metric was minimum mean monthly winter temperature. This metric links estuarine-specific water temperature to large-scale temperature forcing over the scale of the eastern United States [51]–[54] [39] and matches the mechanistic hypothesis of gray snapper range limit [39] with the output of climate models. We used air temperatures from climate models because there is a close association between air temperature and water temperature in shallow estuarine systems [51] [53]–[55]. Further, the scale of the AR4-class climate models is typically 1–2° latitude in the ocean and 2–3° latitude in the atmosphere [26], which is too course to resolve most of the estuarine systems along the east coast of the U.S. To develop the statistical relationship between thermal tolerance metrics and air temperatures, we require daily records of estuarine water temperature (to estimate the thermal tolerance metrics) and annual records of minimum monthly mean winter air temperature.

**Figure 1:**
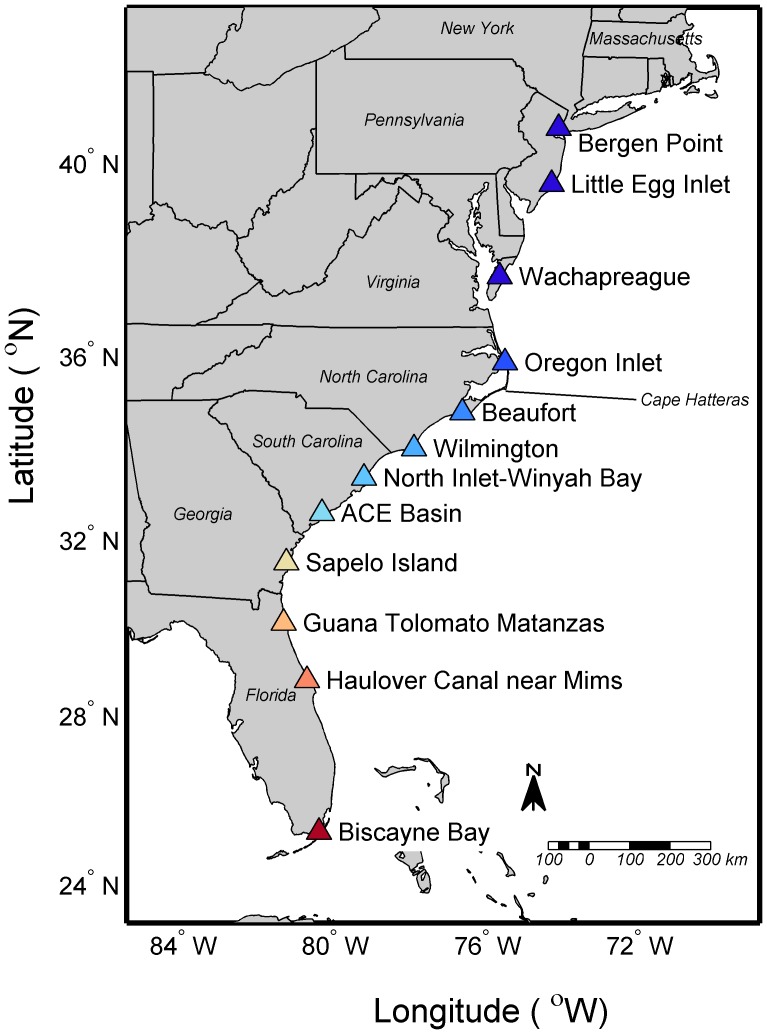
Map showing estuarine locations from which observed temperature records were used and for which projections were made for winter estuarine water temperatures. Color-coding for sites is based on latitude (red more southern, blue more northern). Full list of sites is provided in [39] http://dx.doi.org/10.1016/j.jembe.2012.08.012.

Daily estuarine water temperatures were obtained from 12 sites along the east coast of the United States from Florida to New Jersey (Figure 1). There are numerous records of coastal and estuarine water temperatures along the east coast of the United States [56], [57]. We choose locations based on several criteria: geographic coverage, estuarine location (as opposed to coastal), ongoing data collection, and more than 10 years of data collection. The chosen locations are operated by several organizations and overall have an average of 13 years of daily water temperature observations. Two metrics were calculated from the daily temperature records for each estuary: cumulative degree days below 17°C and minimum daily winter temperature.

Minimum monthly mean winter air temperatures were derived from the NCEP/NCAR reanalysis (see above). For each estuary, the closest continental NCEP/NCAR grid cell was identified. Winter air temperatures over the ocean were warmer than over land and thus we only used grid cells over land to have a consistent comparison between air and estuarine temperatures. In addition, some of the estuaries were proximate to one NCEP/NCAR grid cell, whereas other estuaries were in between grid cells. In this latter case, air temperature values from the two proximate grid cells were averaged for comparison to the estuarine thermal tolerance metrics. After matching the NCEP grid to the location of the estuaries, minimum mean monthly winter temperature (December through March) was extracted from the NCEP/NCAR reanalysis for each of the 12 estuaries.

Annual thermal tolerance metrics (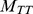) were compared to annual minimum mean monthly winter air temperatures from the NCEP/NCAR reanalysis (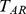) combining data across all estuaries (*j*) and years with data (*y*) with the goal of developing a predictive equation that could be used to project estuarine thermal tolerance metrics from air temperatures and ultimately from climate models. Numerous empirical formulations were evaluated including least squares regression and generalized linear models with Gaussian and Gamma distributions. We determined that the best model was a second order polynomial least squares regression with a square-root transformation of the thermal tolerance metrics:
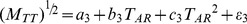
(3)where *a_3_*, *b_3_*, and *c_3_* are parameters estimated with linear least squares regression and 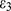 is the unexplained error associated with the regression (eq. 3). The square root transformation addresses the issue that the cumulative degree thermal tolerance metric could not go below zero and the second order polynomial addresses additional nonlinear aspects of the relationship between air temperatures and estuarine water temperatures across estuarine locations.

### Step 3 Thermal Tolerance Metric Projections

The predictive equation developed above (eq. 3) was then used in combination with the mean bias correction (eq. 2) to project the two thermal tolerance metrics 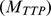 from the projected minimum mean monthly air temperature 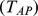 for each climate model (*i*), estuary (*j*), and climate scenario (*k*):
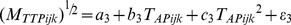
(4)


Thermal tolerance metrics were forecast from 2010 to 2100 for 12 estuaries along the U.S. east coast.

### Step 4 Relationship between Projected Winter Water Temperature and Latitude

Once estuarine thermal tolerance metrics were projected with the climate models, a statistical relationship was derived between the projected metrics and latitude (*Lat*). First, the projected thermal tolerance metrics (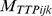) were averaged over three time periods (*t*): 1980–2000, 2040–2060, and 2080–2100. This averaging is necessary because climate models do not produce annual predictions. Due to climate variability, a given year in the model is not expected to match that in nature. Averaging over 20 years reduces the climate variability signal and allows the climate change signal to be examined [26]. The 1980–2000 period was chosen because these years were included in all the 20^th^ century model runs. Second, for each estuary (*j*), climate scenario (*k*), and time period (*t*), the projected thermal tolerance metrics were averaged over the 14 climate models (*i*), which resulted in ensemble average projected thermal tolerance metrics (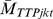) for each estuary, scenario, and time period.

We then developed the statistical relationship between latitude (*Lat*) and the ensemble averaged thermal tolerance metrics (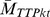) using the data from the different estuaries. Again, numerous empirical formulations were evaluated including least squares regression and generalized linear models with Gaussian and Gamma distributions. We determined that the best model was a first order polynomial least squares regression with a square-root transformation:
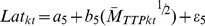
(5)where *a_5_* and *b_5_* are parameters estimated with linear least squares regression and 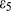 is the unexplained error associated with the regression (eq. 5).

The statistical models represented by equation 5 are generalizations of the projected changes in the latitudinal gradient of thermal tolerances. Changes in latitude with climate change can then be calculated for any specific thermal tolerance value for either metric.

### Step 5 Projections of Northern Range Limits

Cumulative degree days below 17°C (*CDD <17*) was the thermal criteria most closely linked to northern range limit in gray snapper [39] and thus this metric was used to project changes in range of gray snapper. The specific thermal tolerance of gray snapper (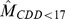 = 210.7±122 [95% confidence interval] days below 17°C) was substituted into regression equation (5) and used to estimate latitude at the thermal criteria for the three climate scenarios (commit, B1, and A1B) and the three time periods (1980–2000, 2040–2060, and 2080–2100).
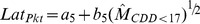
(6)


Rates of change were then calculated (km yr^−1^) from the current period to compare with other studies.

### Step 6 Assessing Uncertainty

Planque et al. [58] reviewed the incorporation of uncertainty into projections of species shifts as a response to climate change. They identified seven types of uncertainty: observational process, conceptual model formulation, numerical model formulation, parameter estimates, model evaluation, spatial and temporal scales, and adaptability of living systems. Within this construct, we focus our analysis of uncertainly on the climate model formulations and parameter estimate uncertainty. We treat the other sources of uncertainty in the discussion. Overall, we assess which aspects of our approach created the most uncertainty as a way to guide future research and to develop more precise projections.

To consider the effect of climate model formulation, emission scenario, statistical downscaling, and parameter estimate uncertainty, we performed the above 5 steps for each climate model, scenario, and time period recalculating equations 3, 5, and 6 and incorporating the error terms ε_3_ and ε_ 5_ and the uncertainty in the thermal tolerance estimates (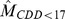). For each model (n = 14), scenario (n = 3), and time period (n = 3), 100 iterations of equations 3, 5, and 6 were performed adding the error terms from a normal probability distribution with a μ = 0 and σ = (model mean squared error)^½^ (for ε_3_ and ε_5_) or σ = thermal estimate standard error (for error on 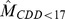). This resulted in 12,600 estimates of the latitude of northern range. These results were analyzed using a general linear model with model, scenario, and time period as categorical variables and the error values as continuous variables. The goal was to determine which factor was responsible for the greatest amount of variability (e.g., uncertainty) in the estimate of northern range. The sums of squares were used to assign a proportion of variance to each factor and these proportions were ranked.

## Results

### Relationship between Winter Water and Air Temperature

**Figure 2:**
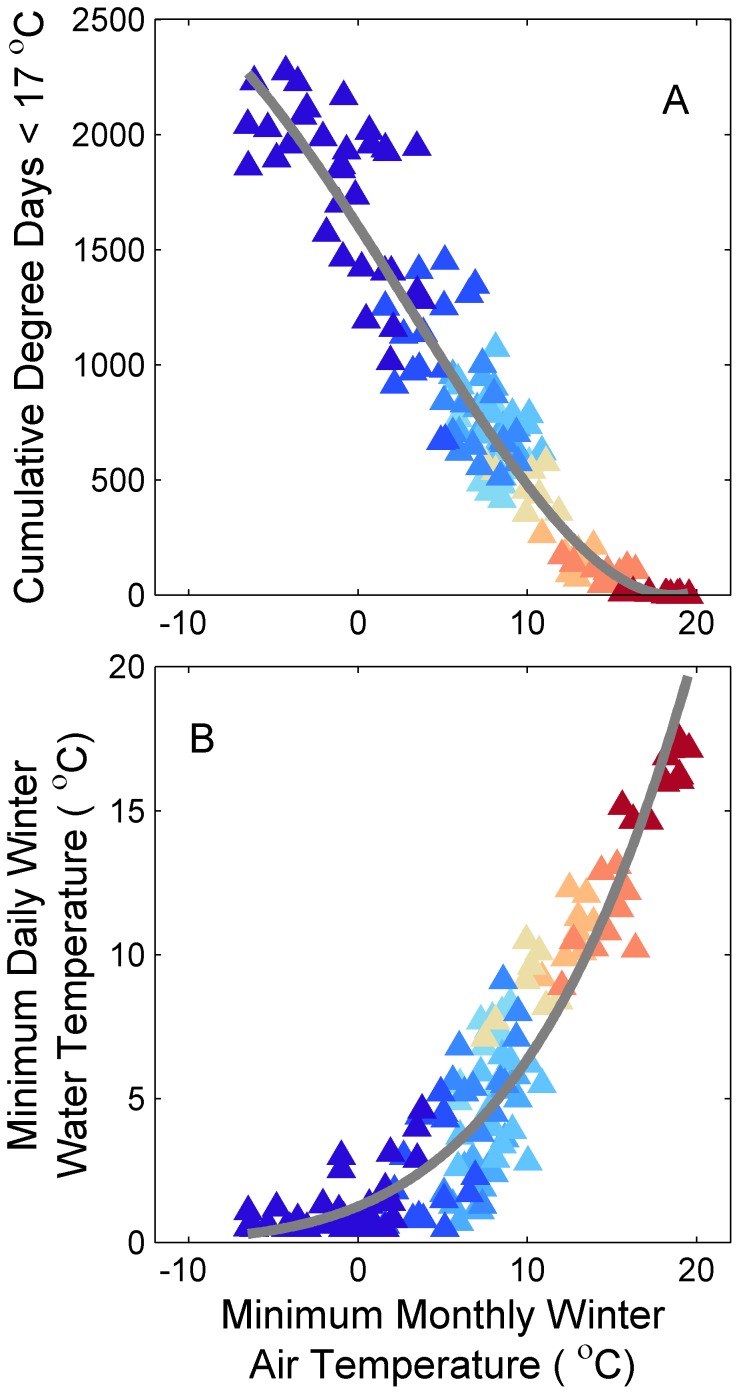
Relations between winter water temperature and air temperature in 12 estuaries along the east coast of the United States. Points represent winter temperatures in a given year in a given estuary. Estuarine water temperatures are expressed as (A) cumulative degree days <17°C and (B) minimum daily winter temperature (Dec-Mar). Air temperature is expressed as minimum monthly mean winter temperature. Gray line represents the least squares regression fit based on eq. 3. Color of the symbol represents the latitude of the source estuary for the water temperature data (see Figure 1).

Over the scale of the east coast of the United States, thermal tolerance metrics could be predicted from air temperatures (Figure 2). Most of the variability in cumulative degree days <17°C could be predicted by minimum mean monthly air temperature (r^2^ = 0.92; Figure 2A). Similarly, most of the variability in minimum daily winter temperature could be predicted by minimum mean monthly air temperature (r^2^ = 0.83; Figure 2B). The resulting predictive equations were then used to estimate thermal tolerance metrics from monthly winter air temperatures derived from the climate models (eq. 4).

### Winter Water Temperature Projections

**Figure 3:**
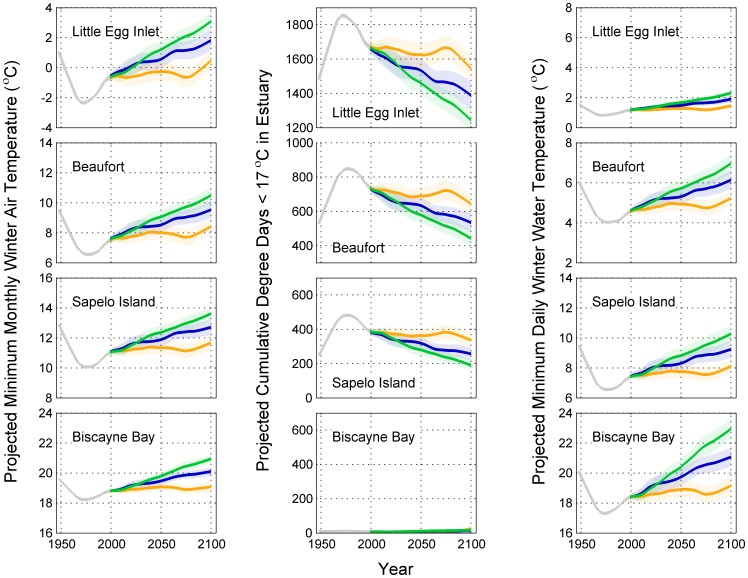
Projections of winter temperature metrics at four estuaries along the east coast of the United States (metrics: minimum mean monthly winter air temperature, cumulative degree days <17°C, and minimum daily estuarine water temperature). Gray line represents observations, orange line represents projections under the commit scenario (350 ppm CO_2_ by 2100), blue line represents projections under the B1 scenario (550 ppm CO_2_ by 2100), and the green line represents projections under the A1B scenario (720 ppm CO_2_ by 2100). A 40 year LOWESS filter (tension = 0.25) of the mean annual projections from 14 general circulation models is displayed (see Table 1). Shading represents standard error around mean. Observed and projected thermal tolerance metrics were blended over the period 2001 to 2010; the blended value in 2005 is 0.5 * observed +0.5 * projected.

Climate model projections indicated increasing minimum mean monthly winter air temperatures over the course of the 21^st^ century (Figure 3, column 1). The magnitude of warming increased with latitude and depended on the climate scenario, with greater atmospheric CO_2_ resulting in greater warming.

Cumulative degree days <17°C were projected to decrease through the 21^st^ century in all estuaries (Figure 3, column 2). The magnitude of decrease increased with latitude and depended on climate scenario, with a greater decrease occurring at higher atmospheric CO_2_ concentrations.

Minimum estuarine daily temperatures were projected to increase through the 21^st^ century in all estuaries (Figure 3, column 3). The magnitude of change decreased with increasing latitude owing to the non-linear relationship with air temperature (see Figure 2). Further, the magnitude of change increased with increasing atmospheric CO_2_.

### Relationship between Projected Winter Water Temperature and Latitude

**Figure 4:**
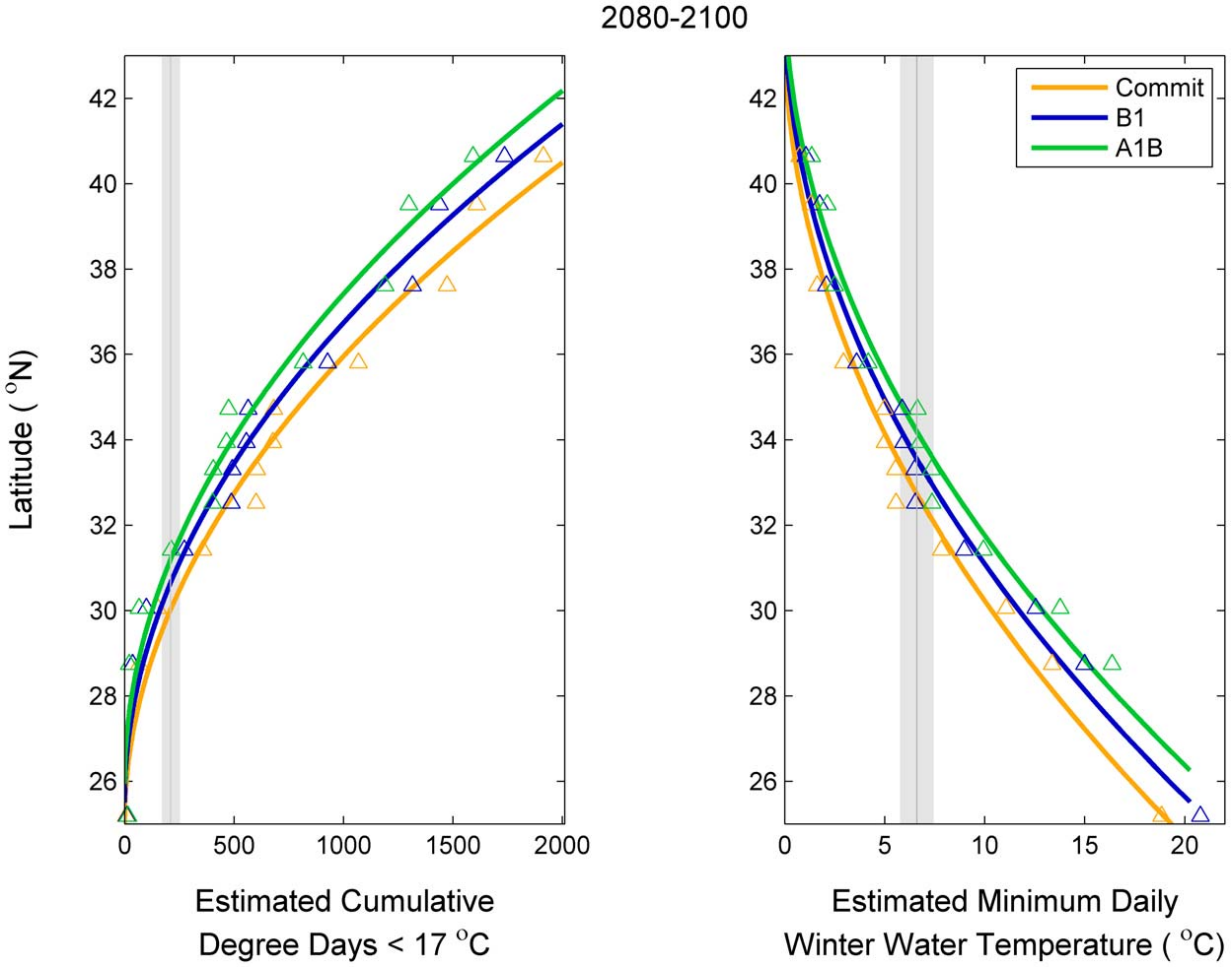
Estimated latitude of thermal tolerance metrics in 2080–2100 under three climate change emission scenarios. The gray line and shading represent the thermal tolerance metric and 95% confidence intervals for gray snapper juveniles as determined from an experimental study [39].

**Figure 5:**
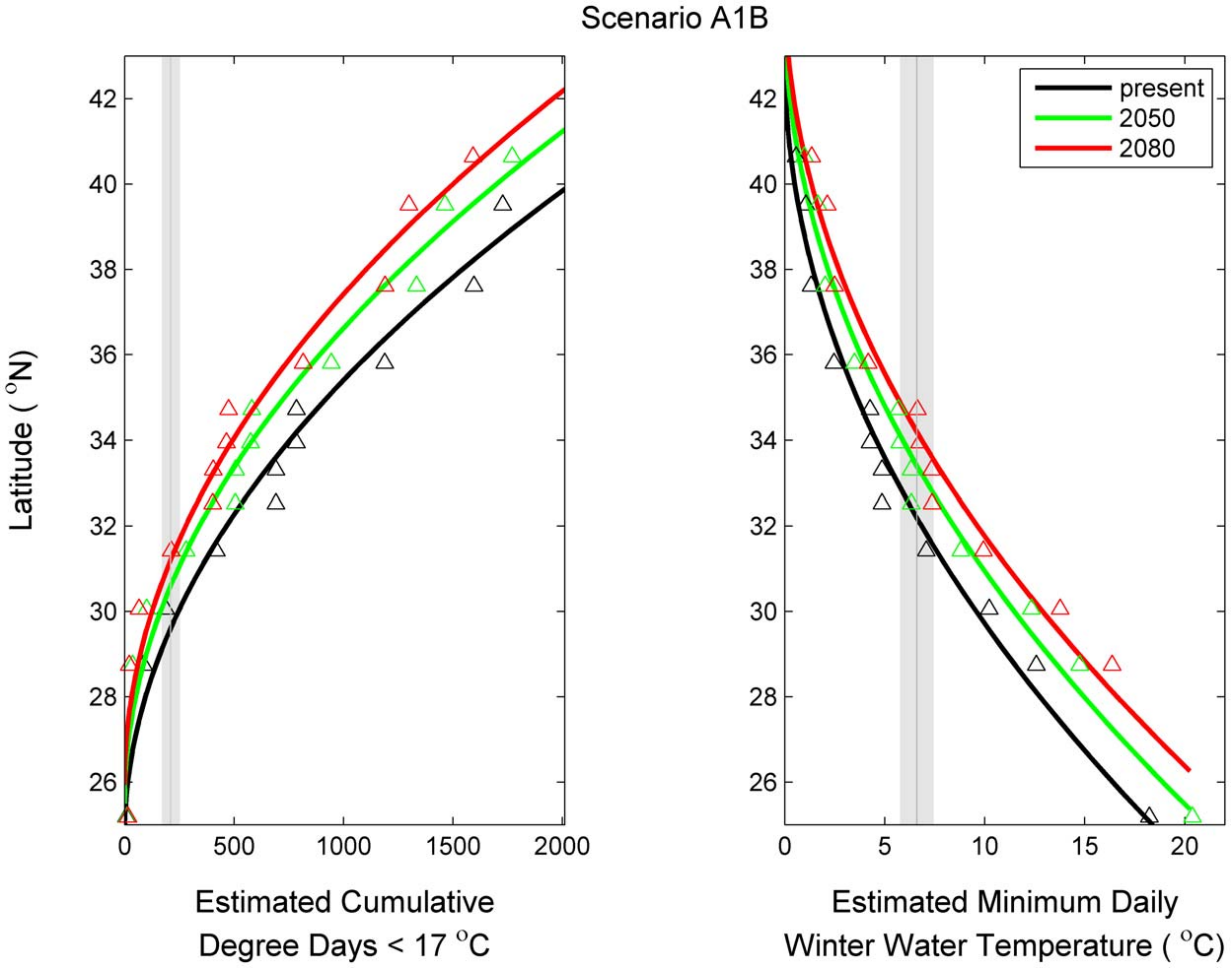
Estimated latitude of thermal tolerance metrics under the A1B emission scenario at three time periods. The gray line and shading represent the thermal tolerance metric and 95% confidence intervals for gray snapper juveniles as determined from an experimental study [39].

Not surprisingly, latitude was well estimated using the two winter water temperature metrics: cumulative degree days <17°C and minimum daily winter temperatures (Figure 4 and 5). The linear models using the square root of thermal tolerance metrics as independent variables (eq. 5) explained on average 97 and 98% across the climate scenarios (commit, B1, and A1B) and time periods (1980–2000, 2040–2060, and 2080–2100). The latitude of a given value of cumulative degree days <17°C increased as the amount of CO_2_ in the atmosphere increased (Figure 4A) and increased into the future (Figure 5A). The same pattern was observed for daily minimum winter temperature. The relation between thermal temperature metrics and latitude may not be continuous; there may be breaks associated with biogeographic breaks (e.g., Cape Canaveral and Cape Hatteras). However, more complex models did not statistically improve the fit and thus, the relatively simple empirical relationship (eq. 5) was used here.

### Projections of Northern Range Limits

**Figure 6:**
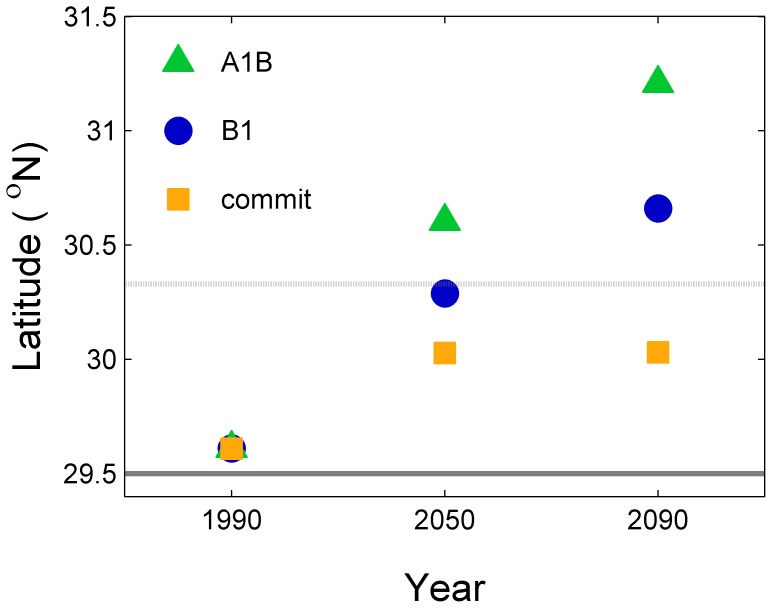
Estimated northern range limit of gray snapper during three time periods and under three emission scenarios. The heavy and light gray lines represent the estimate of current northern range limit and standard error as determined from field observations [39].

Gray snapper range was projected to move northward through time and with increased CO_2_ emissions (Figure 6). The greatest projected northern range was for the period 2080–2100 and the A1B scenario. Under the commit scenario, there is an initial northward movement (0.8 km yr^−1^), but then this movement ceases. Estimated rate of northward shifts were 1.0–1.3 km yr^−1^ under the B1 scenario and 1.7–1.8 km yr^−1^ under the A1B scenario.

### Assessing Uncertainty

**Figure 7:**
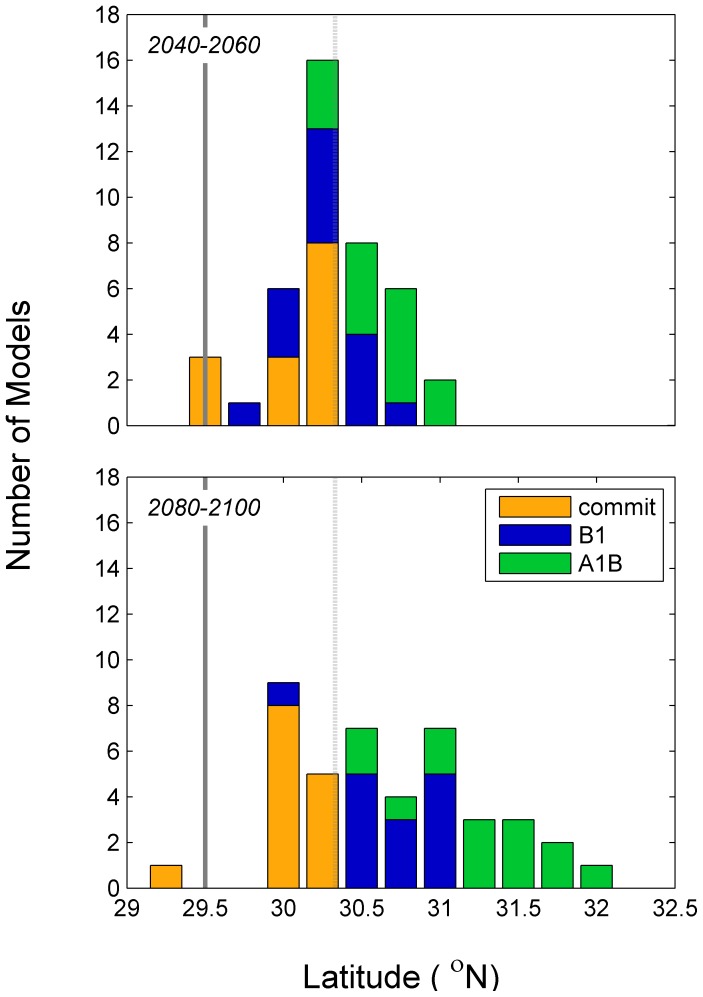
Estimated northern range limit of gray snapper during two time periods and under three emission scenarios. Results for each of the 14 GCMs are provided to present the range of projections that compose the ensemble. The heavy and light gray lines represent the estimate of current northern range limit and standard error as determined from field observations [39].

Most of the variance in the estimate of northern latitude resulted from uncertainty in the thermal tolerance estimate (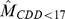) (Table 3; i.e., the variance in CDD <17°C thermal tolerance metric). Uncertainty resulting from the relationship between thermal tolerance and latitude (εe_5_) also contributed a large amount of variance. A relatively small amount of uncertainty was attributed to the climate models themselves; under scenarios B1 and A1B, all individual models projected a poleward range extension (Figure 7). If each scenario and time period is analyzed separately, the average variance attributable to climate models is <0.1%. Thus, a majority of uncertainly is related to the biological parameterization of thermal tolerance (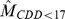) and the estimate of the spatial distribution of the thermal tolerance metrics (eq. 5). There is relatively little uncertainty related to differences among the 14 climate models. Further, there is relatively little uncertainty associated with the method of statistical downscaling (eq. 3).

**Table 3:** Percent variance in estimate of gray snapper northern range attributable to different factors.

Parameter	Percent Variance
Thermal Tolerance Estimate	65.3
Mapping to Latitude	20.8
Unexplained Error	6.0
Time Period	5.6
Scenario	1.8
Model	0.5
Statistical Downscaling	0.0

Value calculated by dividing sums of squares from a general linear model by the total error.

## Discussion

### Projected Poleward Movement

The modeling conducted here projects that adult gray snapper distribution will move northwards over this century. The rate of movement is dependent on CO_2_ emissions, with greater northward shifts projected with increasing CO_2_ emissions. Under the ensemble average and A1B emission scenario, gray snapper are estimated to be distributed half way up the coast of Georgia by 2100 (31.2° N); the most extreme climate model projections estimates that gray snapper will be found up to the Georgia-South Carolina border by the end of the century (31.9° N) (Figure 7).

The rates of poleward movement estimated in our study are broadly consistent with observed and projected changes from other studies. Nye et al. [22] estimated an annual change of 1.5 km yr^−1^ in the center of biomass of fishes on the northeast U.S. continental shelf. These rates of change are lower than the 19 km yr^−1^ observed for marine species in general [59] and lower than the 4.5–5.9 km yr^−1^ projected for marine fishes globally from 2000–2050 [30]. These differences may result from many factors. 1) Our estimates are species specific whereas the estimates cited above are global averages over more than a 1000 fish species and more than 50 marine taxa respectively [30] [59]. 2) Our estimates are regionally specific (east coast of the United States) whereas the estimates cited above are again global in nature [37] [59]. Regional differences in the effect of climate change on marine fishes have been found [37]. Our results also demonstrate regional differences; the decrease in cumulative degree days <17°C is greater further towards the pole (Figure 4 and 5) suggesting greater range extensions for species that are more tolerant of colder winters. 3) The link between air temperature and estuarine temperature may mediate the effect of climate change on coastal fishes. Similar projections for land animals indicate a 0.6 km y^−1^ rate of change [13]. Thus, changes in the ocean may be greater than changes over land [30]; changes in estuarine and coastal systems may be intermediate between the land and ocean. There are other possible explanations, some of which are discussed in more detail below. The overarching conclusion of these studies and other observations are consistent across species and systems; marine species are shifting poleward and will continue to shift poleward in the future. The extent of the poleward shift is dependent in large part on the future of CO_2_ emissions.

**Figure 8:**
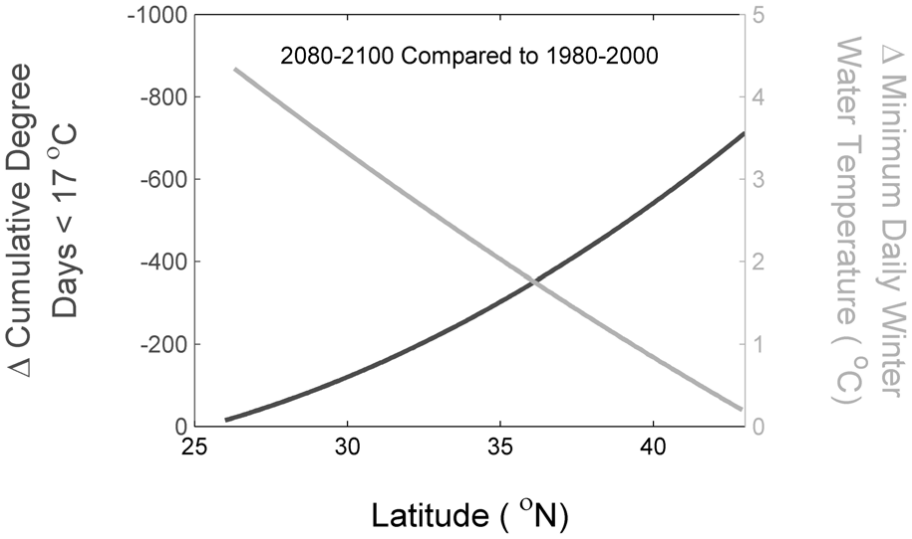
Change in projected cumulative degree days <17°C (dark line) and change in minimum daily temperature (gray line) by latitude under the A1B scenario comparing 1980–2000 and 2080–2100.

Our results suggest a differential rate of winter estuarine warming over the U.S. east coast. Many of the climate models used in our ensemble project increased warming with increased latitude. This is consistent with historic ocean observations, with a greater rate of warming in the northeast U.S. shelf compared to the southeast U.S. shelf [56]. This result is also consistent with the idea of differential warming between the northeast and southeast United States [60]. These results suggest that for species at the northern edge of their range, the more cold-tolerant the species is, the more habitat that will open along the east coast of the U.S. This ‘opening of thermal habitat,’ however, is simply dependent on degree days and does not consider other aspects of habitat. The rate of change in cumulative degree days <17°C is higher at higher latitudes whereas the rate of change of minimum daily temperature is higher at lower latitudes (Figure 8). The implication is at a given latitude, the importance of chronic versus acute thermal tolerances could switch at some point in the future. Thus, there is the potential for a switch in the dominant factor affecting the northern range in gray snapper; currently cumulative degree days appear to be limiting, but at some point in the future minimum daily temperature may become more important. These results emphasize the need to better understand both the physiology and ecology of thermal tolerance as well as the physics of temperature extremes [61]. Higher latitude areas are projected to warm faster, particularly in the northern hemisphere [62], but whether our results are applicable to other temperate marine systems remains unclear.

One novel aspect of this study is the formal assessment of uncertainty in the climate projection of northern range of gray snapper. The full range of factors contributing to uncertainty identified by Planque et al. [58] was not examined, but a number of important factors were considered. Limitations of the observation methodology (observational uncertainty) were not included. A number of observations were used to develop the projections presented here and uncertainty in these observations was not included in the projection model: for example, estuarine temperatures, diver-based adult censuses, and cold-tolerance experiments with juveniles. We are making the implicit assumption that measurement error makes only a small contribution to the overall uncertainty in our projections.

Uncertainty in the conceptual model also was not examined, but a formal hypothesis was presented that can be tested in the future. We hypothesize that northern range of gray snapper is determined by overwinter survival of young-of-the-year related to thermal tolerance. We did not identify a specific mechanism of overwinter mortality (e.g., predation vs. starvation vs. physiology). Our conceptual model is more specific than general niche-based models, in that we identified a specific ontogenetic stage and season in which distribution is determined and we developed specific parameters measuring the physiological boundary of wintertime niche for juvenile gray snapper. It would be interesting in the future to compare projections among a number of niche-based models including the more general models [30] and our more specific model to begin to understand conceptual model uncertainty. It would also be useful be develop this approach using bioenergetic models that would capture the physiological tradeoffs of metabolism, growth, and activity [63].

Uncertainty in the numerical model formulation was evaluated through the use of 14 general circulation models (GCM’s). Each general circulation model simulates the ocean-atmosphere-land system. Overall these models are similar, but differ in numerous details and this ensemble-based approach is becoming the norm in climate projections of living marine resources [26]. We used a bias correction, which is a simple approach. We did not evaluate uncertainty in the model specific bias term but our use of an ensemble of models includes the uncertainty in the bias correction between models. Additionally, other downscaling approaches could be used; we did not evaluate the uncertainty introduced by our choice of using a simple bias correction relative to other techniques. Future studies could examine the magnitude of uncertainty related to these issues. In our evaluation, very little of the overall uncertainty was attributed to the specific GCM and all GCM’s projected an increase in the poleward extent of gray snapper (Figure 7). Thus, we conclude that numerical model formulation contributes only a minimal amount of uncertainty in our specific case. This result is qualitatively similar to Hare et al. [32] in which 14 GCM’s all projected a similar response of Atlantic croaker to climate change.

The dominant source of uncertainty that we were able to evaluate was parameter uncertainty. We included three CO_2_ emission scenarios, each of which contributes a suite of parameters to the GCM’s. We also included uncertainty in our mapping of temperature on latitude and in the thermal limit of gray snapper. Parameter uncertainty is relatively straightforward to include in climate projections since it is often obtained from the statistical error of a parameter estimate [58]. Parameter uncertainty in the estimate of thermal tolerance was the dominant source of uncertainty (Table 3), indicating the need to improve parameter estimates for inclusion in coupled biology-climate models.

Uncertainty in the performance of projection models can result in the uncertain identification of the “best” predictive model [58]. Validation of independent datasets is likely the best form of model evaluation. In the case of gray snapper, our ability to evaluate model performance is limited. The model correctly projects the current northern limit of gray snapper but there is no historical time series of species distribution with which to compare. Further, there is little trend in temperatures along the southeast U.S. [60], so past changes in distribution along the southeast U.S. shelf might be minimal. In the Gulf of Mexico, gray snapper have spread northward as overwintering conditions have become more favorable [23], thus supporting our choice of a model based on overwinter mortality. These comparisons are qualitative and the best evaluation of our model will be revisiting the projections in the future and comparing with observations.

The scale of data, models, and projections also can generate uncertainty [58]. As an example, scaling mismatch between the grain size of environmental variables and distributional data (i.e., species data) can amplify the uncertainties inherent in each of the datasets. We did not include scale uncertainty in our projections, but our parameterization of mapping thermal conditions to latitude was a major source of uncertainty (Table 3). Higher spatial resolution of the observation and models could reduce this uncertainty. We used a simple averaging approach to match temperatures from the 2–3° climate model grid to each specific estuary. Given the large-scale coherence in temperature along the U.S. east coast [54]–[56], this simple routine is likely adequate. However, there is no doubt that higher resolution climate models would reduce the spatial uncertainty of our model. In addition, increasing the spatial resolution of temperature observations could improve the model. This increase in spatial resolution could be achieved by both observations for more estuarine areas along the coast and from mapping temperatures within each specific estuary. There are likely areas of thermal refuge that vary from estuary to estuary [64] [65]–[66]. Quantifying the amount of thermal habitat in each estuary could be used to derive an estimate of total area of overwintering habitat available, which could be relevant to range and population abundance.

The final source of uncertainty identified by Planque et al. [58] is the adaptability of living systems. We assume the thermal tolerance of gray snapper will remain constant into the future, yet adaptation may generate unexpected resilience to climate change [67]. The selective pressures on marine fish can be mitigated by the ability to move [68]–[69], potentially limiting the selective pressures for adaptation. For gray snapper specifically, larvae are dispersed along the entire U.S. east coast. Genetic differences exist between gray snapper from the Gulf of Mexico and the eastern coast of Florida [70]; this pattern is seen in many other fishes as well [71]–[72]. In gray snapper from the Gulf of Mexico, there is evidence for isolation-by-distance [70], so despite the potential for broad larval dispersal, there is the possibility for local genetic differences to arise. Such genetic separation has yet to be evaluated on the southeast coast of the United States. Further, the broader issue of adaptation in thermal tolerance of marine fish needs to be investigated in detail [73].

Our analyses allowed assessment of the relative magnitude of uncertainty generated by different aspects of the forecasting approach. These results suggest that most of the uncertainty can be assigned to the parameter uncertainty related to the thermal tolerance threshold. This emphasizes the need for experimental studies to support the development of ecological climate forecasts. This need has been pointed out by others in much broader context [74] [75]–[76], but it remains important to emphasize that experimental studies are critical to developing and improving the predictions of the effect of climate change on living marine resources.

### Conclusions

Our results indicate that gray snapper along the southeast coast of the United States will spread northward in the coming decades. Larvae are already dispersed well north of the adult range and the northward spread of adults will occur as a result of warming during the winter. This projection is based on the hypothesis that northern range of gray snapper is determined by distribution of overwinter mortality of young-of-the-year [39]. This hypothesis and the resulting projection do not include ecological interactions such as predation and structural habitat requirements. Inclusion of additional factors in a range model will serve to restrict projected range. By analogy the realized niche (determined by ecological interactions) is a subset of the fundamental niche (determined by environmental tolerances) and the forecasts here represent a maximum range extension, not limited by other environmental or ecological processes. These other processes will also be changed by climate, therefore additional complexity incorporating ecological interactions will be needed to develop more complete models of species distributions. In addition, it is important to more fully capture uncertainty in projections, which will serve to communicate the certainty in a projection (e.g., gray snapper will spread northward) and also can be used to identify key areas of research (e.g., improved estimates of thermal tolerance). The major source of uncertainty identified here is the parameter estimate of thermal tolerance, not the climate model, climate scenario, or statistical downscaling approach. This identifies the biological parameterization of coupled climate-biology models as the highest priority in future research to understand the effect of climate change on the distribution and abundance of marine species.
